# Supplementary material for: RAC1B Regulation of TGFB1 Reveals an Unexpected Role of Autocrine TGFβ1 in the Suppression of Cell Motility
Source: Cancers (Basel). 2020 Nov 29;12(12):3570. doi: 10.3390/cancers12123570 (PMC7760153; doi:10.3390/cancers12123570)
Supplement: Supplementary file 1 [file cancers-12-03570-s001.zip › cancers-950540-supplementary/cancers-950540-supplementary R3.pdf]

Supplementary Figure S1

A

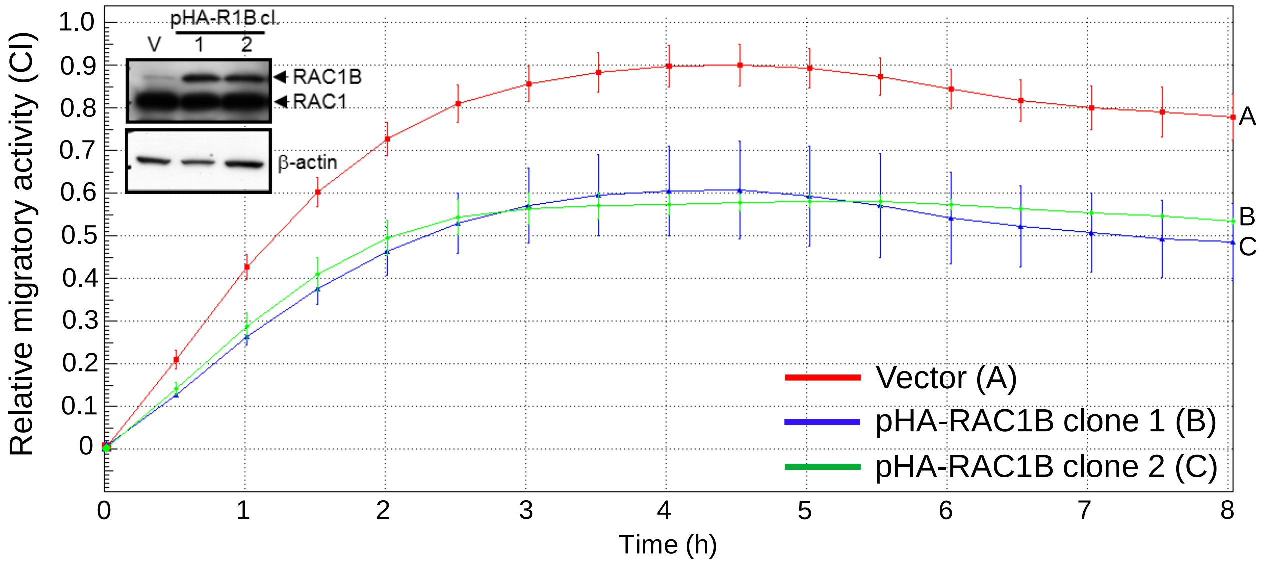

Figure S1A. Effect of RAC1B ectopic overexpression on random cell migration in MDA-MB-231 cells. MDA-MB-231 cells stably expressing empty vector or a HA-tagged version of RAC1B (two different individual clones (cl.)) were subjected to real-time cell migration assay. Data are the means ± SD from 3-4 parallel wells from a representative assay. Inset, immunoblot showing empty vector-transfected control cells (V) and two clones with ectopic expression of HA-RAC1B (pHA-R1B). The anti-Rac1 antibody used recognizes both RAC1 (lower band) and RAC1B (upper band). Equal loading was verified with an antibody to β-actin.

B

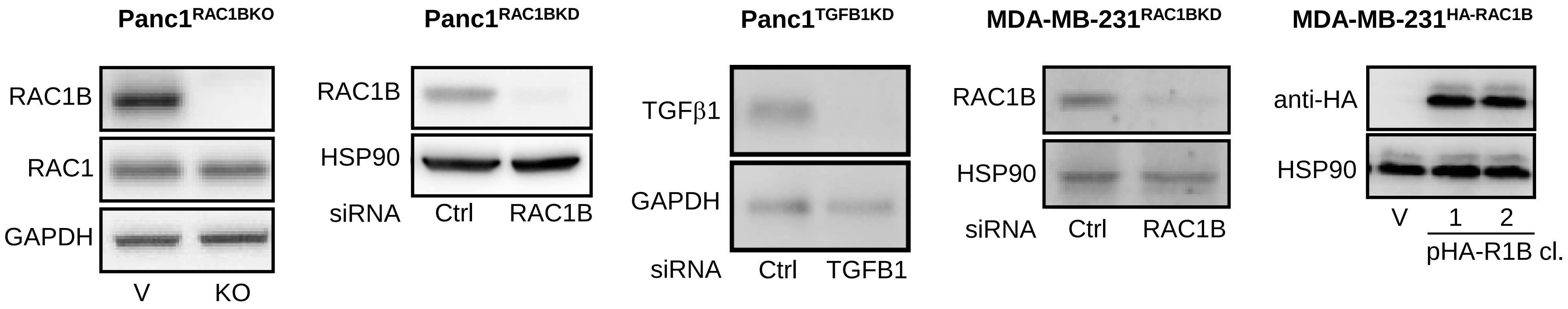

Figure S1B. Verification of KO, KD, or overexpression of RAC1B, TGFB1, or HA-RAC1B, respectively, in Panc1 and MDA-MB-231 cells. cl., clone.

Supplementary Figure S2

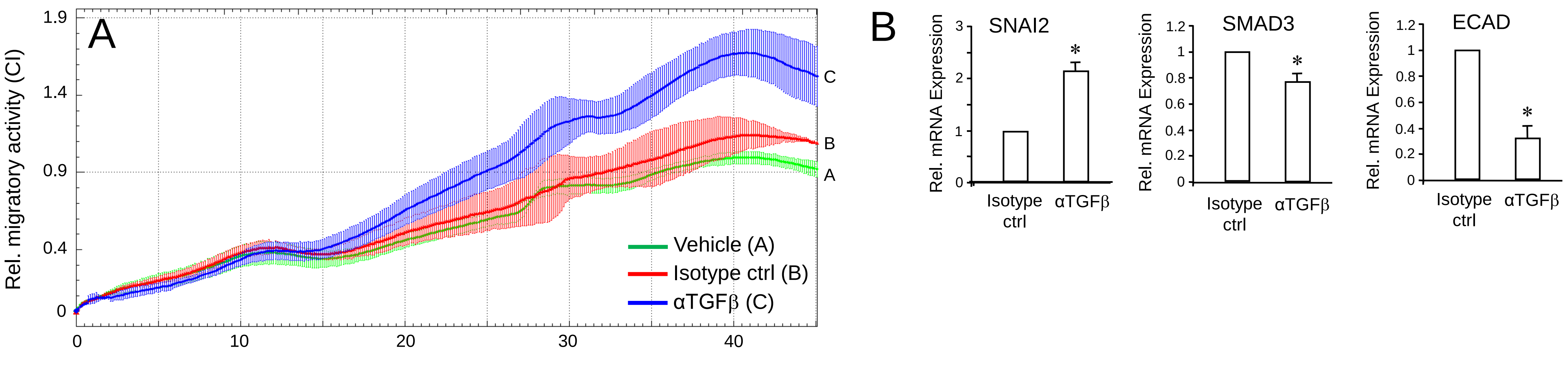

Figure S2. Effects of antibody-mediated neutralization of secreted autocrine TGFβ on cell motility of Panc1 cells. (**A**) Panc1 cells (5,000 cells/well in 50 µl of growth medium containing 1% FBS) were subjected to real-time cell migration assay in the absence or presence of either vehicle, IgG1 isotype control antibody (isotype ctrl, 50 µg/ml) or pan-anti-TGFβ1/2/3 antibody (αTGFβ, 50 µg/ml). Results shown are representative of three assays. Data represent the mean ± SD of three parallel wells. In panel (**A**), differences between curves C and A were first significant at 18:00 and between curves C and B at 24:00, and remained so until completion of the assay. (**B**) In parallel to the migration assays, cells in separate wells were treated under the same conditions with isotype ctrl or αTGFβ for 24 h and processed for RNA isolation and qPCR analysis of SNAI2, SMAD3, ECAD, and RAC1. SNAI2 rather than SNAI1 was chosen here because in an orthotopic mouse model of human pancreatic cancer SNAI2 was mainly expressed at the tumor's invasive front, associated with a repressed ECAD expression in migrating tumor cells. This suggests that SNAI2 contributes to the maintenance of the malignant phenotype and promotion of cell motility [Hotz, B.; Arndt, M.; Dullat, S.; Bhargava, S.; Buhr, H.J.; Hotz, H.G. Epithelial to mesenchymal transition: expression of the regulators snail, slug, and twist in pancreatic cancer. *Clin. Cancer Res*. **2007**, *13*, 4769-4776]. No changes between αTGFβ and isotype ctrl treatment were seen for RAC1 mRNA (not shown). Data represent the mean ± SD of triplicate wells. The asterisks indicate statistical significance relative to isotype ctrl treated cells. *, $p < 0.05$.

Supplementary Figure S3

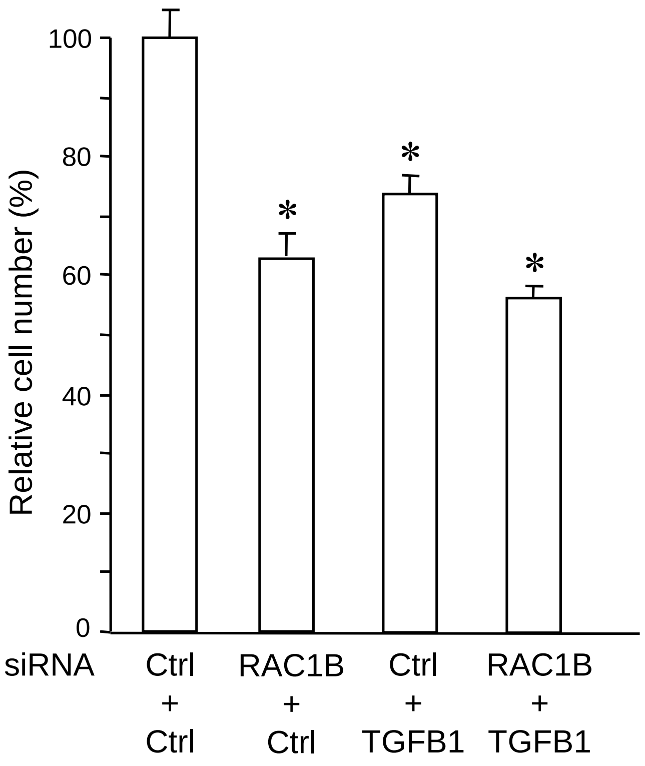

Figure S3. SiRNA-mediated knockdown of RAC1B or TGFB1 inhibits basal proliferation. Panc1 cells were transiently transfected with 50 nM each of control siRNA (Ctrl), RAC1B siRNA, or TGFB1 siRNA, followed by another 24-h incubation in medium supplemented with 1% FBS. Cells were then detached and counted manually with a Neubauer chamber. Data represent the mean ± SD of four wells processed in parallel. Asterisks indicate significance relative to the Ctrl+Ctrl siRNA-transfected cells. The assay is representative of three experiments with very similar results. Aliquots of cells were tested for successful inhibition of siRNA targets by immunoblot analysis (see Figure 3B, inset).

Supplementary Figure S4

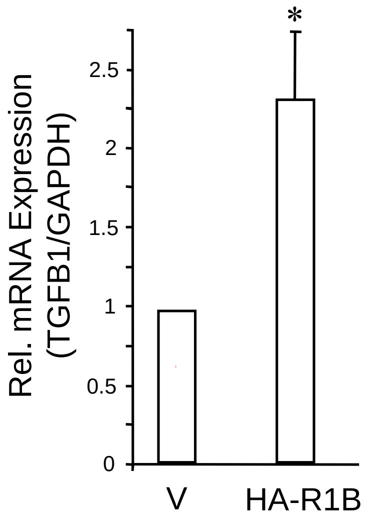

Figure S4. Effect of ectopic overexpression of RAC1B on mRNA levels of TGFB1 in Panc1 cells. Panc1 cells stably expressing empty vector (V) or a HA-tagged version of RAC1B (HA-R1B) were sampled from continuous cultures at regular intervals and subjected to RNA isolation. Total RNA was reverse transcribed and subjected to qPCR of TGFβ1 and GAPDH. Data represent the normalized mean ± SD from three samples. The asterisk (*) denotes a significant difference relative to the vector control.

Supplementary Figure S5

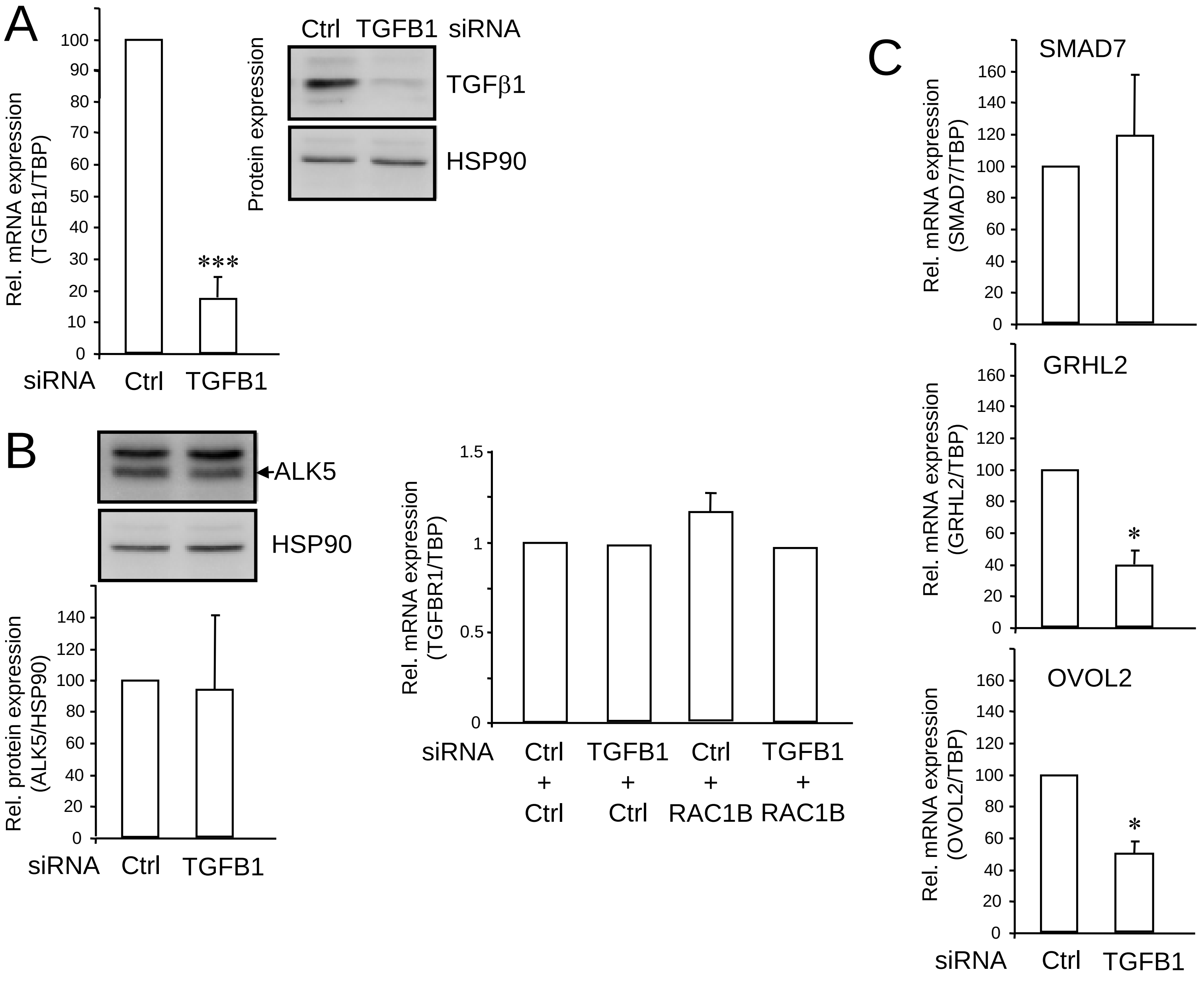

Figure S5. Effect of TGFB1 or RAC1B knockdown on genes associated with TGFβ signaling or epithelial differentiation. Panc1 cells transiently transfected twice with 50 nM of either a control (ctrl) siRNA or a TGFβ1-specific siRNA were processed 48 h later for qPCR analysis and immunoblot analysis of (**A**) TGFB1, (**B**) ALK5/TGFBR1 or (**C**) qPCR analysis of SMAD7, GRHL2 or OVOL2. Data represent the mean ± SD from at least three different transfections. Immunoblot based verfication of RAC1B and TGFB1 siRNA-mediated KD for the samples shown in (**B**) can be found in Figure 3. The asterisks indicate a significant difference relative to controls (***, $p < 0.001$, $n = 4$; *, $p < 0.05$, $n = 3$).

Supplementary Figure S6

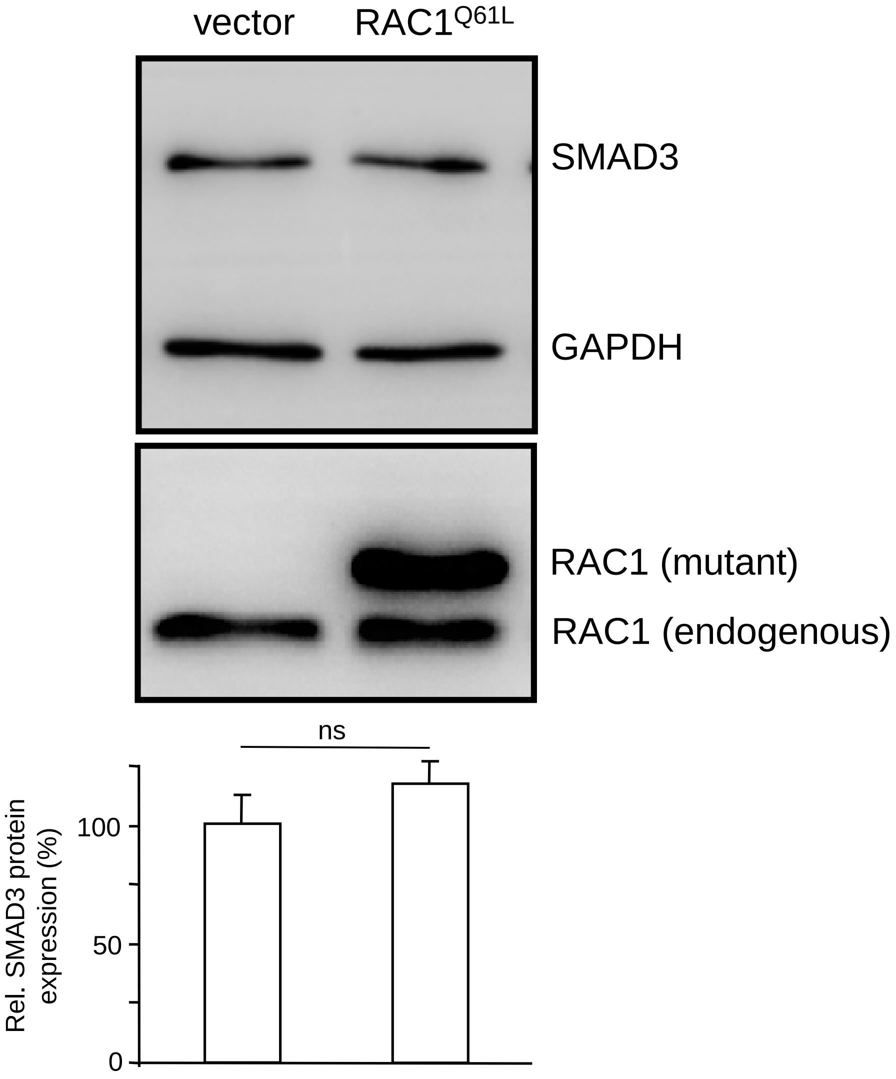

Figure S6. Effect of ectopic expression of the constitutively active RAC1 mutant, RAC1^{Q61L}, on SMAD3 protein abundance in Panc1 cells. Panc1 cells transiently transfected with an expression vector for MYC-RAC1^{Q61L} (RAC1^{Q61L}) or empty pRK5 vector (vector) were lysed 48 h after transfection and fractionated by SDS-PAGE, blotted and successively incubated with antibodies to SMAD3, RAC1, and GAPDH as a loading control. The Rac1 antibody recognizes both the endogenous RAC1 (lower band) and the mutant RAC1 (upper band), which due to the MYC tag has a lower electrophoretic mobility. The graph shows quantification of densitometric readings from three transfections/blots relative to vector controls set at 100% (mean ± SD, n=3), ns, non-significant.

Supplementary Figure S7: Uncropped blot of Figure 3B, inset

Western blot from Figure 3B, inset

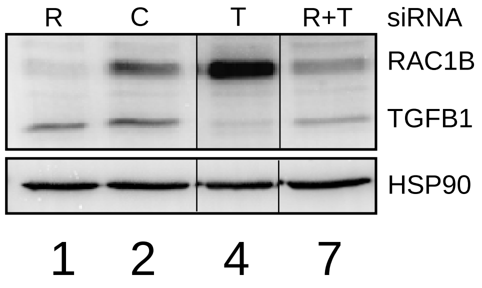

Uncropped blot

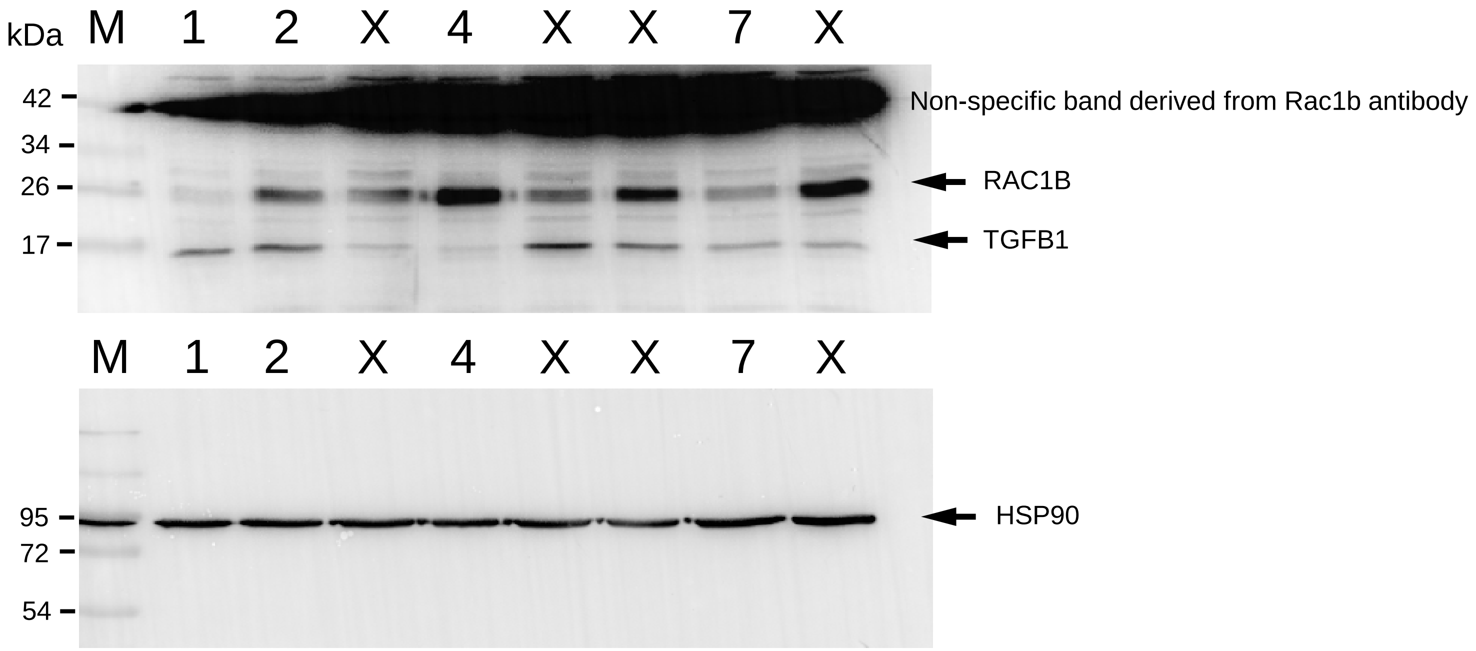

M = molecular weight marker (SM1841, Fermentas/Thermo Fisher Scientific)
X = irrelevant lanes that were removed
