# Supplementary material for: RAC1B Regulation of TGFB1 Reveals an Unexpected Role of Autocrine TGFβ1 in the Suppression of Cell Motility
Source: Cancers (Basel). 2020 Nov 29;12(12):3570. doi: 10.3390/cancers12123570 (PMC7760153; doi:10.3390/cancers12123570)
Supplement: Supplementary file 1 [file cancers-12-03570-s001.zip › cancers-950540-supplementary/Uncropped blots_Figures 3-5_completed.pdf]

Uncropped blots from Figure 3A (inset)

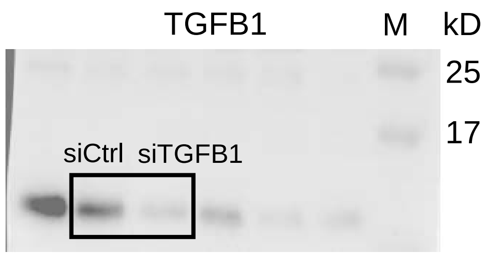

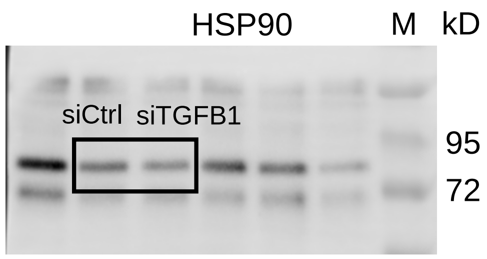

Figure legend: M = molecular weight marker, siCtrl = control siRNA, siTGFB1 = TGFB1 siRNA

Uncropped blots and densitometric readings from Figure 4

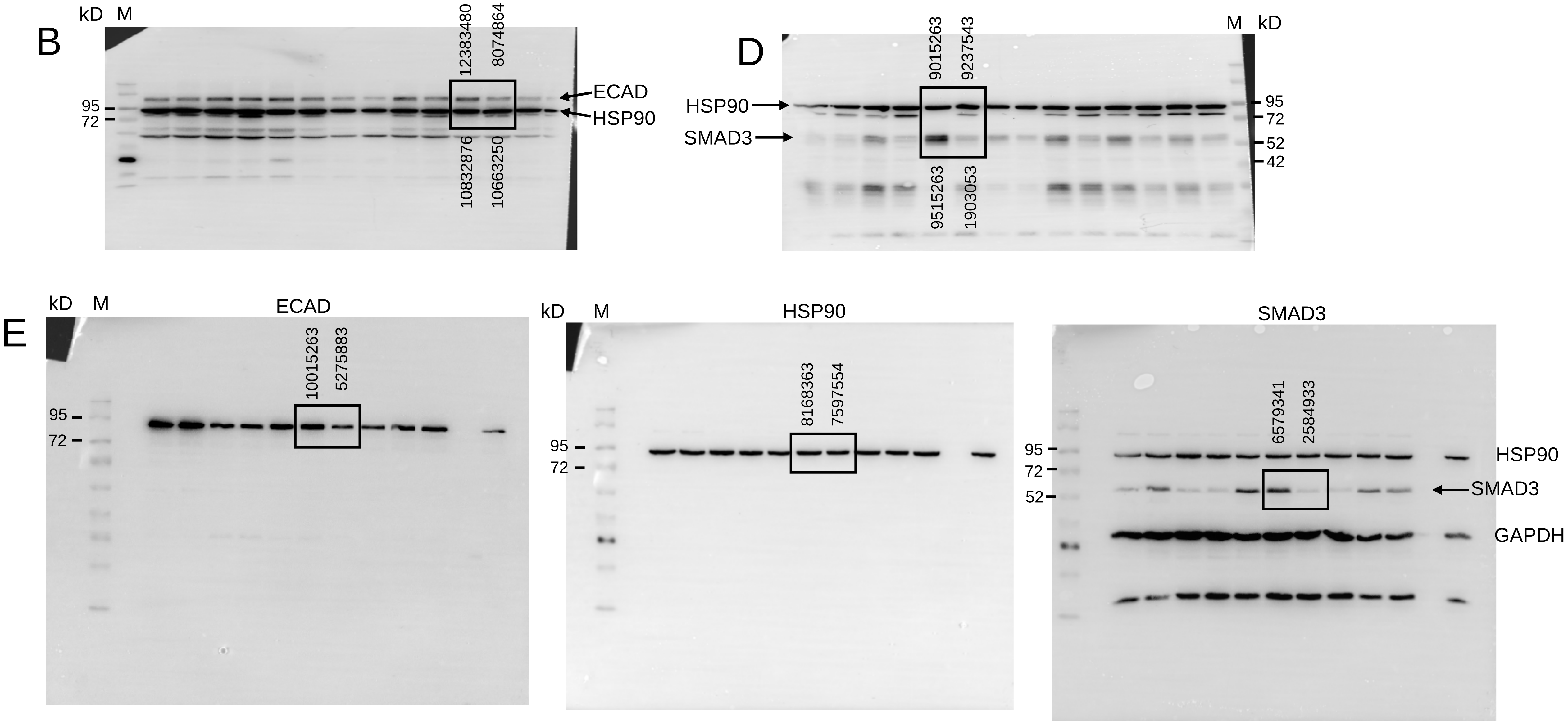

Figure legend: M = molecular weight marker; the numbers above or below the bands denote signal intensities/densitometric readings

Uncropped SMAD3 immunoblots from Figure 5 (insets)

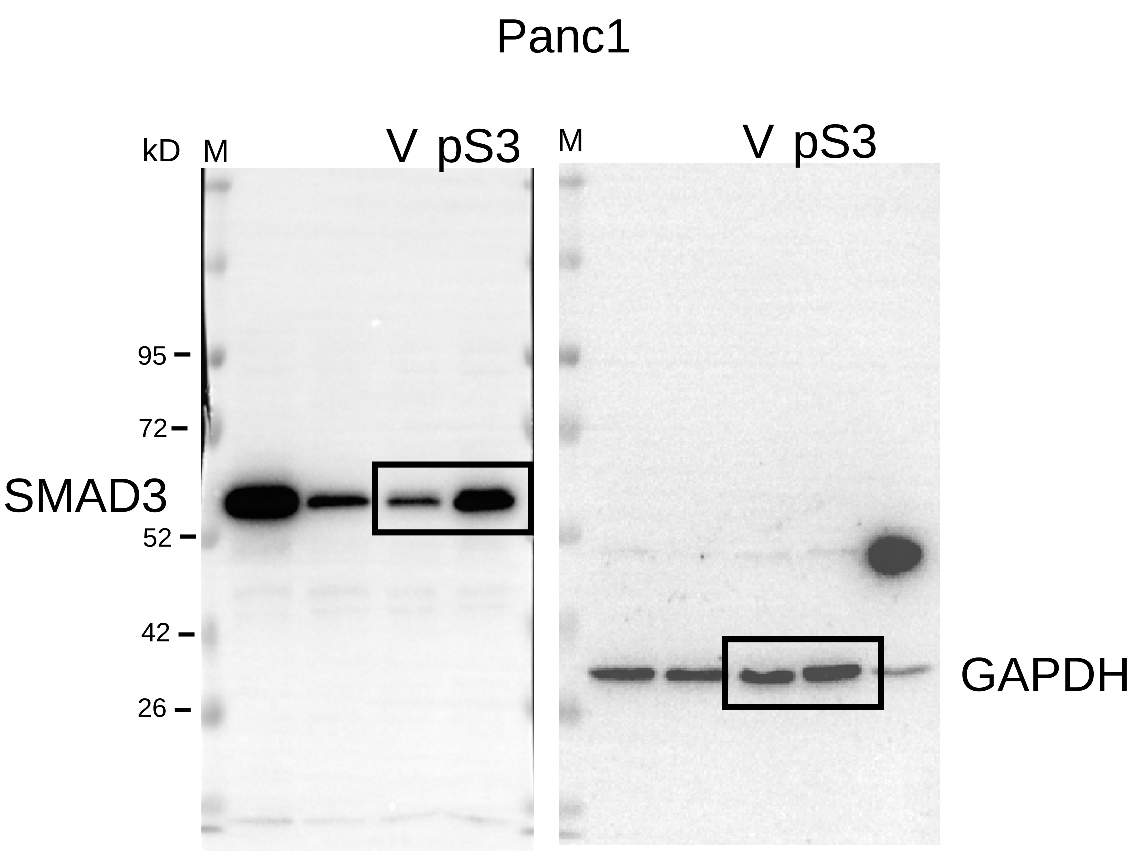

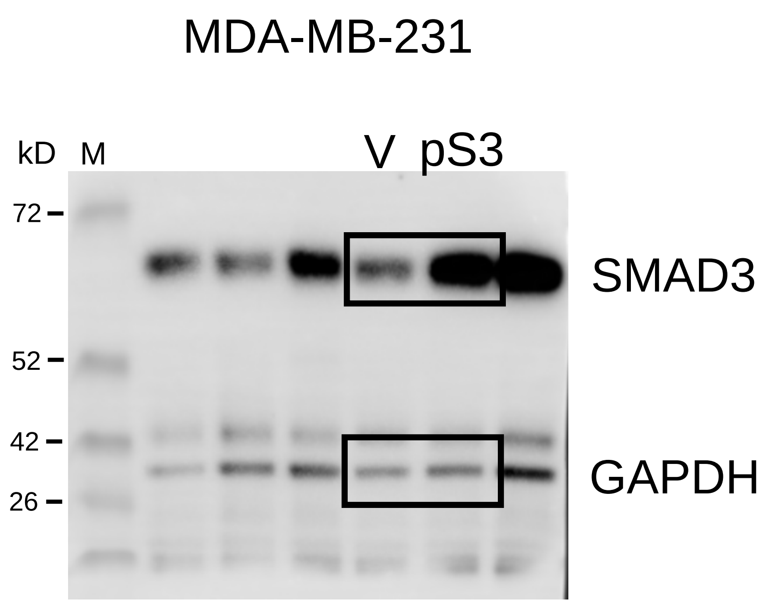

Figure legend: M = molecular weight marker, V = empty vector, pS3 = SMAD3 expression vector

Uncropped TGFβ1 immunoblots from Figure 5 (insets)

Panc1

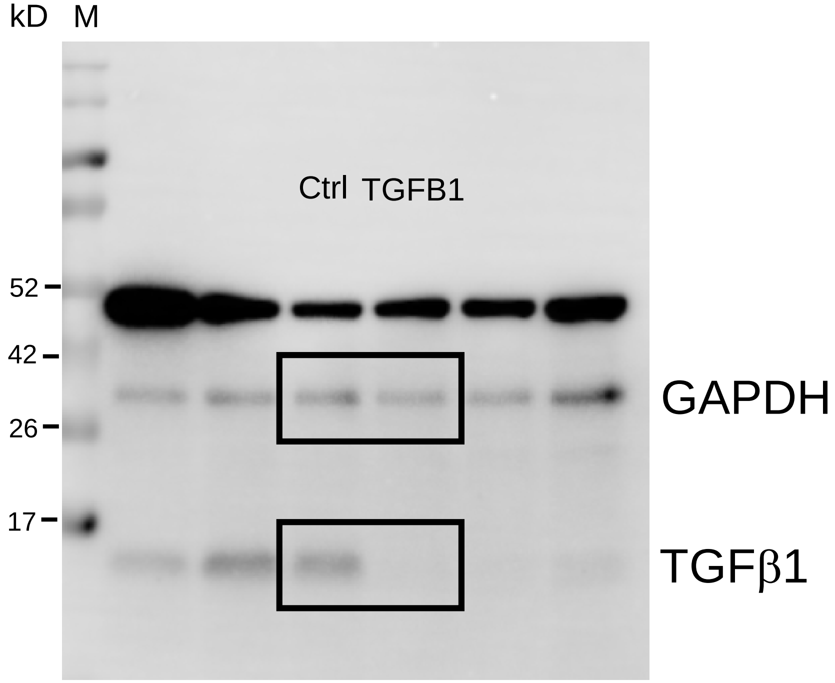

MDA-MB-231

Short exposure
(for TGFB1 signal)

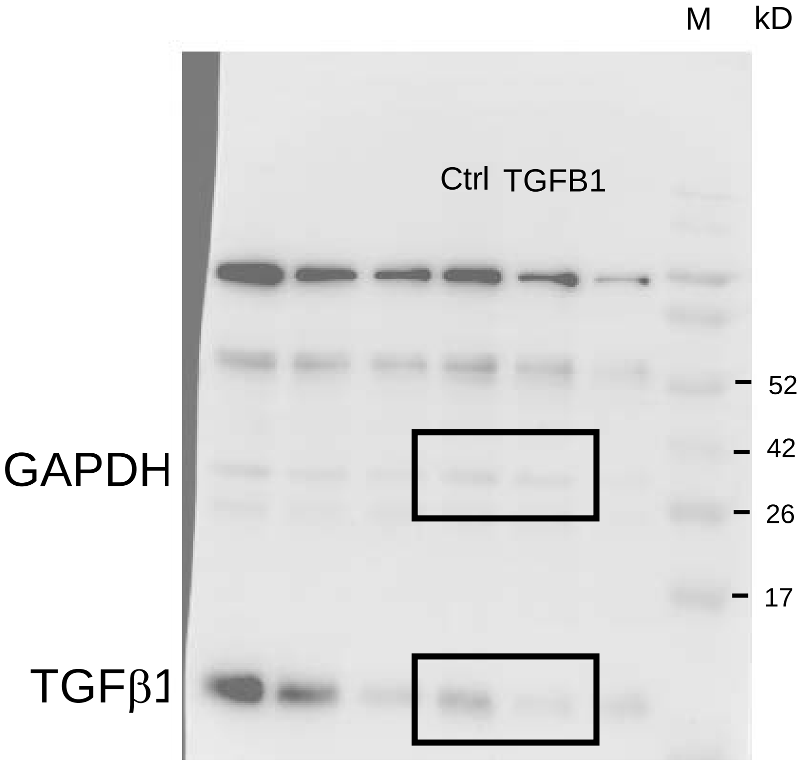

MDA-MB-231

Long exposure
(for GAPDH signal)

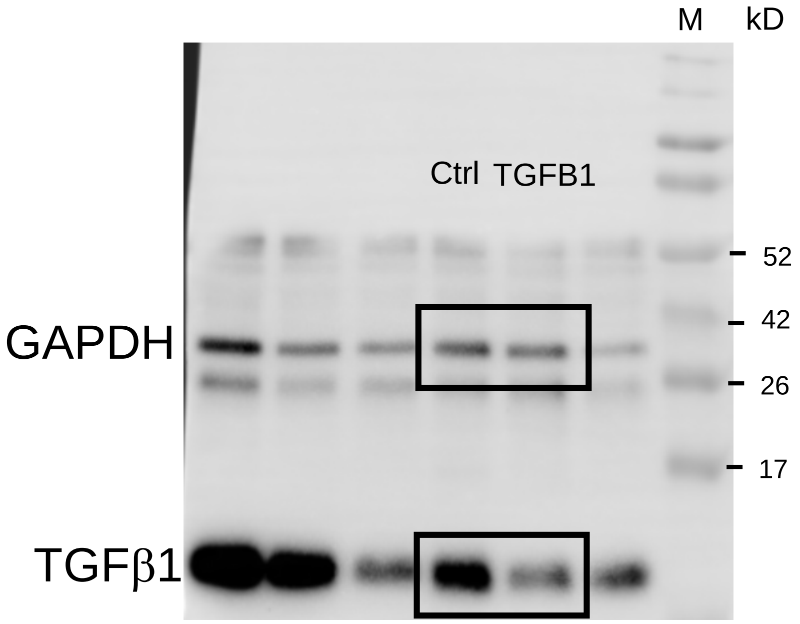

Figure legend: M = molecular weight marker, Ctrl = control siRNA, TGFB1 = TGFB1 siRNA
