# Supplementary material for: RAC1B Regulation of TGFB1 Reveals an Unexpected Role of Autocrine TGFβ1 in the Suppression of Cell Motility
Source: Cancers (Basel). 2020 Nov 29;12(12):3570. doi: 10.3390/cancers12123570 (PMC7760153; doi:10.3390/cancers12123570)
Supplement: Supplementary file 1 [file cancers-12-03570-s001.zip › cancers-950540-supplementary/Table S1_Primers for PCR.doc]

**Supplementary Table S1** Primers used for qPCR

| **Designation** | **Sequence (5’→3’)** |
| --- | --- |
| ALK5-sense | GCGACGGCGTTACAGTGTTTCTGC |
| ALK5-antisense | ATGGTGAATGACAGTGCGGTTGTGG |
| E-cadherin-sense | TCTTCCCCGCCCTGCCAATC |
| E-cadherin-antisense | GCCTCTCTCGAGTCCCCTAG |
| GAPDH-sense | TTGCCATCAATGACCCCTTCA |
| GAPDH-antisense | CGCCCCACTTGATTTTGGA |
| GRHL2-sense | GGGGAAGAGCAACGAGTGG |
| GRHL2-antisense | GCGCTGGTCGTCTTTGAGA |
| INHBA-sense | CCTCCCAAAGGATGTACCCAA |
| INHBA-antisense | CTCTATCTCCACATACCCGTTCT |
| OVOL2-sense | ACAGGCATTCGTCCCTACAAA |
| OVOL2-antisense | CGCTGCTTATAGGCATACTGC |
| RAC1 exon 3b-antisense | GGCAATCGGCTTGTCTTTGCCC |
| RAC1-sense | ACCATGCAGGCCATCAAGTGTGTGG |
| RAC1-antisense | TTACAACAGCAGGCATTTTCTCTTC |
| SLUG-sense | ATATTCGGACCCACACATTACCT |
| SLUG-antisense | GCAAATGCTCTGTTGCAGTGA |
| SMAD3-sense | TGGACGCAGGTTCTCCAAAC |
| SMAD3-antisense | CCGGCTCGCAGTAGGTAAC |
| SMAD7-sense | GGAAGATCAACCCCGAGCTG |
| SMAD7-antisense | TTGGGAATCTGAAAGCCCCC |
| SNAIL-sense | CTGCTCCACAAGCACCAAGAGTC |
| SNAIL-antisense | CCAGCTGCCCTCCCTCCAC |
| TBP-sense | GCTGGCCCATAGTGATCTTT |
| TBP-antisense | CTTCACACGCCAAGAAACAG |
| TGFβ1-sense | cccAGCATCTGCAAAGCTC |
| TGFβ1-antisense | GTCAATGTACAGCTGCCGCA |
| TGFβ2-sense | cagcacactcgatatggacca |
| TGFβ2-antisense | cctcgggctcaggatagtct |
| TGFβ3-sense | acttgcaccaccttggacttc |
| TGFβ3-antisense | ggtcatcaccgttggctca |
